# Effectiveness of influenza vaccines in preventing acute cardiovascular events within 1 year in Beijing, China

**DOI:** 10.1038/s41541-024-00969-y

**Published:** 2024-09-28

**Authors:** Yuan Ma, Feng Lu, Luodan Suo, Wei Li, Jie Qian, Tianqi Wang, Min Lv, Jiang Wu, Weizhong Yang, Moning Guo, Juan Li, Luzhao Feng

**Affiliations:** 1 School of Population Medicine and Public Health, Chinese Academy of Medical Sciences & Peking Union Medical College, Beijing, China; 2 State Key Laboratory of Respiratory Health and Multimorbidity, Beijing, China; 3 Key Laboratory of Pathogen Infection Prevention and Control (Peking Union Medical College), Ministry of Education, Beijing, China; 4 Department of Medical Records, Peking Union Medical College Hospital, Chinese Academy of Medical Sciences & Peking Union Medical College, Beijing, China; 5 Beijing Municipal Health Big Data and Policy Research Center (Beijing Institute of Hospital Management), Beijing, China; 6 Beijing Center for Disease Prevention and Control, Beijing Research Center for Preventive Medicine, Beijing, China; 7 Center for Applied Statistics and School of Statistics, Renmin University of China, Beijing, China

**Keywords:** Epidemiology, Cardiovascular diseases

## Abstract

Controversies persist about the protective effects of vaccines against acute cardiovascular events. Using electronic medical records from hospitals and influenza vaccine administration data in Beijing, China, we studied individuals vaccinated between January 1, 2016, and December 31, 2018, who experienced at least one acute cardiovascular event within two years. A self-controlled case series design calculated the relative incidence (RI) and 95% confidence interval (CI) of acute cardiovascular events within one year after vaccination. Among 1647 participants (median age: 65 years, 38.43% female), the risk of events 29–365 days post-vaccination was 0.76 times the baseline level (RI: 0.76; 95% CI: 0.68–0.84). The protective effect was more pronounced in younger participants (*P* = 0.043) and those without cardiovascular history (*P* < 0.001), while acute respiratory infection (*P* = 0.986) and vaccination frequency (*P* = 0.272) had no impact. Influenza vaccines offer protection against acute cardiovascular events for at least one year, suggesting potential for cardiovascular disease prevention.

## Introduction

Cardiovascular disease ranks first among the top ten causes of death globally^1^ and is the leading cause of total mortality in both urban and rural residents in China^2^. Increasing evidence suggests that acute respiratory infections can increase the risk of acute cardiovascular diseases^3,4^. Influenza vaccination is an effective intervention to prevent acute respiratory infections and reduce their severity^5^, it could also prevent related cardiovascular complications^6^. Previous studies have suggested a relationship between influenza vaccination and reduced risk of acute cardiovascular events such as myocardial infarction, stroke, and intracerebral hemorrhage^7,8^. Some scientific associations and health regulatory agencies have recommended influenza vaccination to prevent the occurrence of cardiovascular events^5,9^.

Although numerous studies have shown an association between influenza vaccination and reduced risk of cardiovascular events, there is still controversy regarding the results for different populations and types of cardiovascular diseases^10–12^. This may be related to study design, characteristics of the study population, and frequency of vaccine administration. In observational studies, unknown confounding bias and indication bias may affect the reliability of the research; randomized controlled trials have rigorously defined the included populations, leading to potentially different results from real-world research. Additionally, many previous studies exploring the protective effect of vaccines on acute cardiovascular events did not consider the acute respiratory infection as a confounding factor since it is a risk factor for acute cardiovascular events. Self-controlled case series designs use individuals as their own controls to better control for non-time-varying confounders and estimate effects during different exposure periods^13,14^. However, there are currently few studies based on Chinese population using this type of research design to explore the protective effects of influenza vaccines on cardiovascular events.

Acute respiratory infections could increase the risk of acute cardiovascular events, regardless of whether individuals have underlying cardiovascular diseases^15^. Influenza vaccination, as a cost-effective measure to prevent acute respiratory infections, may have significant potential in preventing cardiovascular diseases. In order to more effectively reduce the risk of cardiovascular diseases, this study aims to provide more evidence on the association between influenza vaccination and cardiovascular events, and clarify how this association changes over time.

## Methods

### Ethics

The study was approved by the Medical Ethics Committee of the Chinese Academy of Medical Sciences and Pecking Union Medical College, Beijing, China (CAMS&PUMC-IEC-2022-019, March 14 2022) and was conducted according to the Declaration of Helsinki. The need for informed consent was waived by the ethics committee because the study utilized de-identified hospitalization data collected as part of routine clinical practice. The waiver was granted based on the minimal risk posed to participants by the research and impracticality of obtaining consent due to the retrospective nature of the study.

### Data source

The study utilized influenza vaccine vaccination records in Beijing from January 1, 2016 to December 31, 2018 obtained from the Beijing Center for Disease Prevention and Control. Additionally, electronic medical records for hospitalizations in Beijing from 2012 to 2020 were linked from the Beijing Municipal Health Big Data and Policy Research Center, a citywide hospital admission surveillance system in Beijing, China^16,17^. Due to incomplete vaccination data for residents from other provinces, the study population was limited to permanent residents of Beijing (identified based on basic medical insurance information in hospitalization data).

### Study design

The self-controlled case series study (SCCS) was conducted. The exposure factor was influenza vaccination, and the outcome of interest was acute cardiovascular events. The time of influenza vaccination was taken as the start point of the observation period, and hospital admission time of acute cardiovascular events was considered as the occurrence time of the disease. The observation period was defined as follows: from the day of influenza vaccination (day 0) until 730 days thereafter. Since protective antibodies from influenza vaccine typically develop 2–4 weeks after vaccination^18,19^, and influenza vaccine is usually administered once a year, this study defined the exposure period as follows: from day 29 after influenza vaccination to 365 days thereafter. The control period was defined as the observation period excluding the exposure period; i.e., 0–28 days and 366–730 days after influenza vaccination. This method for dividing time intervals is similar to that used in a previous study^20^.

**Fig. 1:**
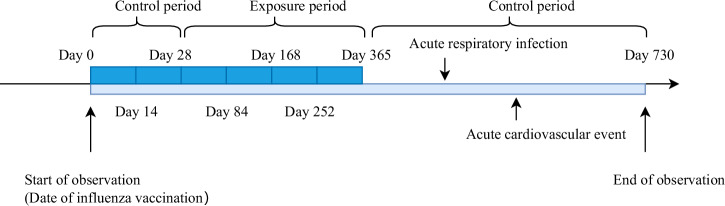
The observation period of the self-controlled case series study. A 2-year observation period was established, with the first year after influenza vaccination as the exposure period and the second year after influenza vaccination as the control period.

According to previous research, acute respiratory infections are risk factors for acute cardiovascular events^21,22^; therefore, this study also considered acute respiratory infections occurring after vaccine administration but before outcome events as confounding exposure factors with an exposure window set at post-infection day 0-365^4^. The schematic diagram of the observation period is shown in Fig. 1.

**Fig. 2:**
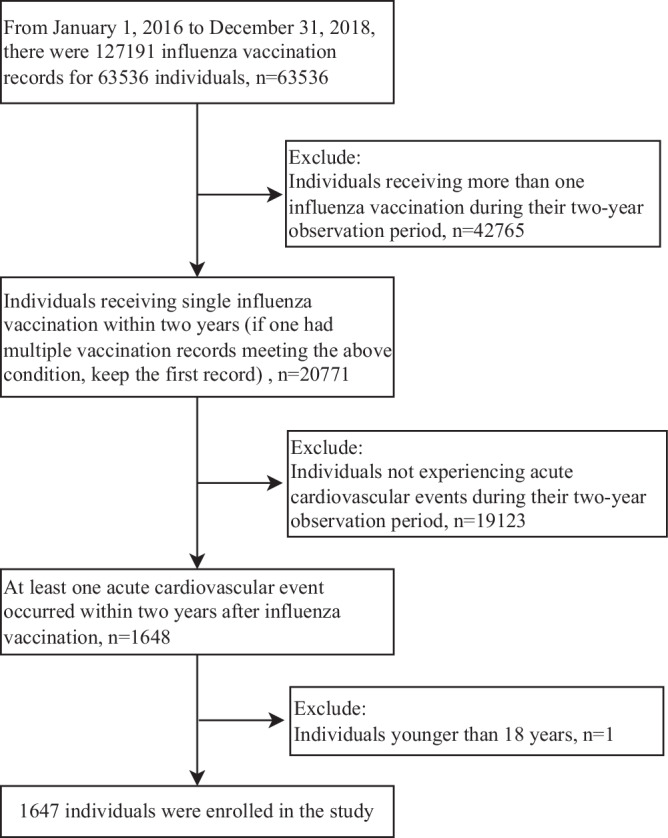
Flow chart for inclusion and exclusion of participants in the study. Participants were individuals who had been vaccinated between January 1, 2016, and December 31, 2018, and had experienced at least one acute cardiovascular event within two years.

In order to minimize potential confounding factors, such as the impact of COVID-19 vaccines, this study focused on analyzing hospital records dated prior to December 31, 2020. Consequently, the administration of influenza vaccinations was restricted to dates before December 31, 2018 to ensure a complete two-year observation period for each individual in the self-controlled case series analysis. Given that influenza vaccination is typically received annually, it was assumed that the protective effect of influenza vaccines would diminish to baseline levels in the second year if individuals did not receive another influenza vaccination during the two-year observation period. Inclusion criteria were: (1) receipt of influenza vaccination between January 1, 2016 and December 31, 2018, without receiving subsequent influenza vaccinations within two years; (2) occurrence of at least one acute cardiovascular event within two years after receiving influenza vaccination. Exclusion criteria were: (1) retention of the first record if an individual had multiple vaccination records meeting the above conditions; (2) exclusion of individuals aged below 18 years. The inclusion and exclusion flowchart is shown in Fig. 2.

For patients who experienced multiple acute cardiovascular events during the observation period, due to potential associations between these events, this study considered the time of occurrence of the first acute cardiovascular event during the observation period as the time point for analysis.

### Exposure and outcomes

The exposure factor was influenza vaccination. China has licensed trivalent (IIV3) and quadrivalent inactivated influenza vaccines (IIV4), including split-virus vaccine, whole-virus vaccines, and subunit vaccine. The specific types of vaccines utilized during the study seasons were shown in Supplementary Table 1. All licensed influenza vaccines contain strains recommended for the northern hemisphere by the World Health Organization (https://www.who.int/teams/global-influenza-programme/vaccines/who-recommendations; http://www.chinacdc.cn/en/). The Supplementary Table 2 presented the influenza vaccine strains, circulating strains, predominant circulating strains, and the concordance between vaccine strains and circulating strains for the 2015–2016 to 2018–2019 influenza seasons.

The outcome event was the acute cardiovascular event, which was identified through discharge diagnosis codes from the International Classification of Diseases, Tenth Revision (ICD-10). Acute cardiovascular events include acute myocardial infarction, stroke, acute myocarditis, acute pericarditis, and other acute cardiovascular diseases. Chronic or subacute diseases such as cerebral infarction (I63.903) and chronic ischemic heart disease (I25) were not included. Detailed disease codes are provided in Supplementary Table 3.

Regarding the vaccination status of influenza vaccine, if a patient’s influenza vaccination record is not matched, it indicates that the patient has not been vaccinated against influenza or has been vaccinated outside of Beijing City. Since this study limited the study population to patients with Beijing medical insurance, the possibility of receiving vaccinations outside of Beijing is low. Therefore, this study considers patients who did not have a matched influenza vaccination record as not having received an influenza vaccine.

### Other factors

Acute respiratory infection was considered another exposure factor due to its intricate association with acute cardiovascular events^21,22^. In this study, the causative agents of acute respiratory infection could not be determined due to unavailable data for laboratory diagnosis. Therefore, the scope of acute respiratory infection in this study includes not only influenza infections but also acute upper respiratory tract infections (ICD-10: J00-J06) and lower respiratory tract infections (ICD-10: J09-J18, J20-J22), which were identified through discharge diagnosis codes from the International Classification of Diseases, Tenth Revision (ICD-10). This study also took into account the seasonal factors, which we divided into four groups: spring (March 21–June 21), summer (June 22–September 22), autumn (September 23-December 21), and winter (December 22–March 20 of the next year). The medical history information of the study population was obtained from previous hospital records, specifically all hospitalization records from 2012 to the onset of the first acute cardiovascular event during the observation period. The medical history information includes cardiovascular diseases (ischemic heart disease and stroke), hypertension, hyperlipidemia, chronic lower respiratory diseases, ulcers, diabetes, kidney disease, liver disease, and cancer. The ICD-10 diagnostic codes for these diseases can be found in Supplementary Table 3.

### Statistical methods

Basic characteristics of participants were described according to the type of acute cardiovascular events. Continuous variables and categorical variables were described using median (quartiles) and frequency (percentage), respectively. Wilcoxon rank-sum test and chi-square test were used for inter-group differences comparison. In addition, the study also provided a characterization and comparison of patients with different cardiovascular disease histories.

This study conducted an analysis of overall acute cardiovascular events and their subtypes (myocardial infarction, ischemic stroke, hemorrhagic stroke, and others). The analysis of subtypes of acute cardiovascular diseases was based on patients with a discharge diagnosis that only included that type of acute cardiovascular disease; patients with multiple types of events occurring simultaneously were excluded. If a patient had multiple hospitalizations for acute respiratory infections within 45 days, the multiple hospitalizations were considered as one episode of acute respiratory infection, and the time of the first admission was taken as the onset time of the disease. The same data processing method was applied to acute cardiovascular events.

We utilized the standard SCCS model and adjusted for acute respiratory infection factors and seasonal effects to calculate the relative incidence (RI) and 95% confidence interval (CI) of acute cardiovascular events during the exposure period (vaccination days 29–365) compared to baseline (vaccination days 0–28, 366–730), as well as RIs of acute cardiovascular events during different time windows within the exposure period (29–84 days, 85–168 days, 169–252 days, 253–365 days). The study also investigated the protective effect of influenza vaccination on acute cardiovascular events within one year among individuals with different cardiovascular disease histories, and its variation over time.

This study conducted a stratified analysis of the protective effect of influenza vaccine, using age, gender, underlying diseases, and occurrence of acute respiratory infections as stratification factors. Likelihood ratio tests were used to explore potential interactions between influenza vaccine administration and these factors. Additionally, to investigate the impact of multiple inactivated influenza vaccine (IIV) immunizations on the prevention of acute cardiovascular events, we categorized individuals based on whether they had received another influenza vaccination within one year prior to the observation period. If an individual had received another influenza vaccination within one year before the current vaccination, they would be classified as having received multiple vaccinations; otherwise, they would be classified as having received a single vaccination. Since only data for influenza vaccination records after September 1, 2015 are available, the earliest start time for the observation period was set to one year later, specifically September 1, 2016.

To ensure the robustness of the results, sensitivity analyses were performed: (1) not adjusting for acute respiratory infections; (2) building models for different influenza seasons separately; (3) excluding patients who experienced both acute respiratory infections and acute cardiovascular events during the same hospitalization; (4) excluding patients who died from acute cardiovascular events during hospitalization. Furthermore, we extended the observation period to three years to investigate the impact of IIV immunity beyond 1 year. The exposure period was defined as 29–730 days after vaccination, and the control period was defined as 0–28 days and 731–1095 days after vaccination. Only individuals who received the influenza vaccination between January 1, 2016, and December 31, 2017, and did not receive any subsequent vaccinations within three years were included in the analysis.

Data analysis was conducted using SAS software (version 9.4), R software (version 4.2.3), and RStudio (version 2023.12.0). The SCCS model was constructed using the “SCCS” package in R. All statistical tests were two-sided with a significance level set at 0.05.

## Results

### Characteristics of participants

As shown in Table 1, a total of 1647 participants were included in this study. Among them, there were 209 (10.67%) patients with myocardial infarction, 600 (42.62%) with ischemic stroke, 68 (2.99%) with hemorrhagic stroke, and 16 (2.13%) with two or more types of the aforementioned acute cardiovascular events, while 754 (41.60%) had other acute cardiovascular events. The median age (Q1–Q3) of participants was 65.00 (56.00–73.00) years. Males accounted for 61.57% and females accounted for 38.43%. During the observation period and at the time of acute cardiovascular events occurrence, a total of 13.05% patients experienced acute respiratory tract infections. In the overall study population, 75.47% had a history of cardiovascular disease. Detailed basic information of participants is provided in Table 1. To demonstrate the timing of influenza vaccination in relation to cardiovascular events among participants, a centered plot^14^ is also drawn (Supplementary Fig. 1).

**Table 1:** Characteristics of participants grouped by types of acute cardiovascular events

	Total events (*N* = 1647)	Myocardial infarction (*N* = 209)	Ischemic stroke (*N* = 600)	Hemorrhagic stroke (*N* = 68)	Two or more types^a^ (*N* = 16)	Others (*N* = 754)
Age (Years), median (Q1–Q3)	65.00 (56.00–73.00)	63.00 (52.00–74.00)	65.50 (57.00–74.00)	58.50 (49.50–73.00)	68.50 (60.00–79.00)	65.00 (57.00–73.00)
Age group (Years), *n* (%)						
<60	547 (33.21)	83 (39.71)	195 (32.50)	36 (52.94)	4 (25.00)	229 (30.37)
≥60	1100 (66.79)	126 (60.29)	405 (67.50)	32 (47.06)	12 (75.00)	525 (69.63)
Sex, *n* (%)						
Male	1014 (61.57)	148 (70.81)	394 (65.67)	46 (67.65)	11 (68.75)	415 (55.04)
Female	633 (38.43)	61 (29.19)	206 (34.33)	22 (32.35)	5 (31.25)	339 (44.96)
Length of hospital stay (Days), median (Q1–Q3)	8.00 (6.00–12.00)	8.00 (6.00–12.00)	10.00 (8.00–13.00)	13.00 (10.00–19.00)	12.50 (10.00–16.00)	7.00 (4.00–10.00)
Acute respiratory infection occurred^b^, *n* (%)	215 (13.05)	28 (13.40)	74 (12.33)	10 (14.71)	3 (18.75)	100 (13.26)
Underlying diseases, *n* (%)						
Cardiovascular diseases	1243 (75.47)	201 (96.17)	278 (46.33)	19 (27.94)	10 (62.50)	735 (97.48)
Chronic lower respiratory infection	250 (15.18)	20 (9.57)	95 (15.83)	9 (13.24)	1 (6.25)	125 (16.58)
Hypertension	1273 (77.29)	137 (65.55)	475 (79.17)	59 (86.76)	14 (87.50)	588 (77.98)
Hyperlipidemia	1401 (85.06)	185 (88.52)	538 (89.67)	26 (38.24)	15 (93.75)	637 (84.48)
Ulcer	138 (8.38)	20 (9.57)	48 (8.00)	15 (22.06)	0 (0.00)	55 (7.29)
Diabetes	572 (34.73)	66 (31.58)	221 (36.83)	10 (14.71)	4 (25.00)	271 (35.94)
Kidney disease	77 (4.68)	10 (4.78)	29 (4.83)	1 (1.47)	0 (0.00)	37 (4.91)
Liver diseases	513 (31.15)	50 (23.92)	172 (28.67)	18 (26.47)	6 (37.50)	267 (35.41)
Cancer	48 (2.91)	6 (2.87)	12 (2.00)	1 (1.47)	1 (6.25)	28 (3.71)

^a^“Two or more types” indicates to the occurrence of at least two types of diseases in the three categories of myocardial infarction, ischemic stroke and hemorrhagic stroke.

^b^Occurring acute respiratory infection before the first acute cardiovascular event during the observation period.

### Protective effect of influenza vaccines on acute cardiovascular events

**Fig. 3:**
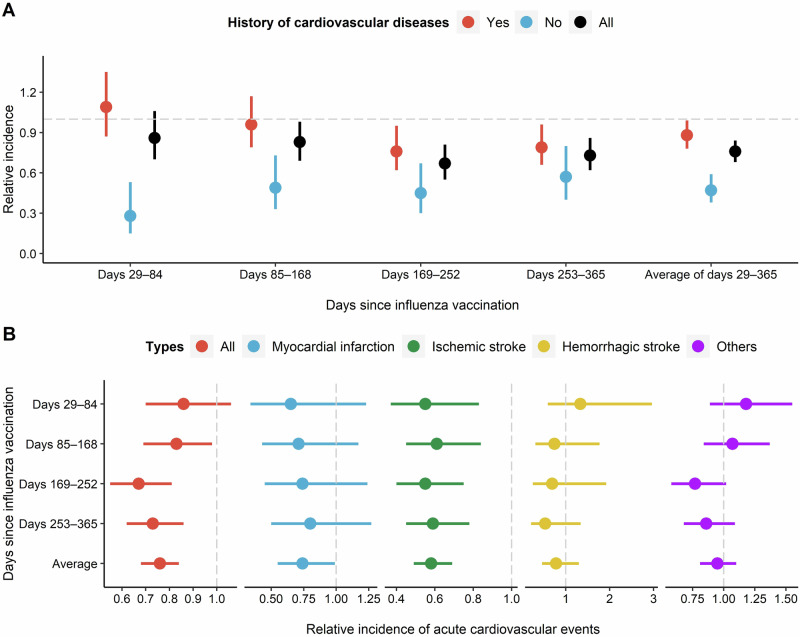
Relative incidence of acute cardiovascular events within one year following influenza vaccination. **A** Relative incidence for all acute cardiovascular events among participants with different status of underlying cardiovascular diseases. **B** Relative incidence for different types of acute cardiovascular events.

The protective effect of influenza vaccination on acute cardiovascular events within 1 year is shown in Fig. 3 and Supplementary Table 4. The risk of acute cardiovascular events occurring 29–365 days after influenza vaccination was 0.76 times the baseline level (0–28 days and 366-730 days post-vaccination) with a RI of 0.76 and a 95% CI of 0.68–0.84. The RIs and 95% CIs for days 29–84 being 0.86 (0.70–1.06), days 85-168 being 0.83 (0.69–0.98), days 169–252 being 0.67 (0.55–0.81), and days 253–365 being 0.73 (0.62–0.86).

As shown in Fig. 3 and Supplementary Table 4, in individuals with a history of cardiovascular disease, the RI and its 95% CI of overall acute cardiovascular events for days 29–84, days 85–168, days 169–252, and days 253–365 were 1.09 (0.87–1.35), 0.96 (0.79–1.17), 0.76 (0.62–0.95), and 0.79 (0.66–0.96) respectively; while in individuals without a history of cardiovascular disease, the RI and its 95% CI for different time periods were 0.28 (0.15–0.53), 0.49 (0.33–0.73), 0.45 (0.30–0.67), and 0.57 (0.40–0.80). The average RI and its 95% CI for individuals with a history of cardiovascular disease and those without cardiovascular disease at days 29–365 were 0.88 (0.78–0.99) and 0.47 (0.38–0.59), respectively.

### Protective effect against different types of acute cardiovascular events

The influenza vaccine has a protective effect on both ischemic stroke and myocardial infarction events within one year (Fig. 3 and Supplementary Table 4). From day 29 to 365 after vaccination, the average risk of ischemic stroke and myocardial infarction decreased by 42% and 26%, respectively compared to the baseline period, with RIs and their 95% CIs being 0.58 (0.49–0.69) and 0.74 (0.55–0.99). Taking ischemic stroke as an example, the RI and its 95% CI from days 29 to 84, days 85 to 168, days 169 to 252, days 253 to 365 were 0.55 (0.37–0.83), 0.61 (0.45–0.84), 0.55 (0.40–0.75), and 0.59 (0.45–0.78) respectively. For other types of acute cardiovascular events, due to the small sample size, the confidence intervals are all wide (Fig. 3 and Supplementary Table 4).

### Stratification analysis

Stratification factors, including age (<60 years, ≥60 years), gender, history of cardiovascular disease, chronic lower respiratory tract disease, hypertension, hyperlipidemia, diabetes, kidney disease, liver disease, cancer prior to the observation period, acute respiratory infection, and the vaccination frequency were considered (Table 2). The protective effect of influenza vaccine is greater in individuals under 60 years old (RI: 0.65, 95% CI: 0.55–0.78) than those aged 60 and above (RI: 0.82, 95% CI: 0.72–0.93). Furthermore, it was found to be more effective in individuals without a history of cardiovascular disease (RI: 0.48, 95% CI: 0.38–0.60) in comparison to those with such a history (RI: 0.87, 95% CI: 0.78–0.98). The study also revealed better protection in individuals without a history of chronic lower respiratory tract diseases (*P* = 0.002) or kidney disease (*P* = 0.002). Additionally, evidence did not show any impact on the protective effect of influenza vaccine from acute respiratory infections (*P* = 0.986).

**Table 2:** Stratification analysis for the association between influenza vaccines and acute cardiovascular events^a^

	No. of events (*N* = 1674)	Relative incidence (95% CI)	*P* value
Age, years			0.043
<60	547	0.65 (0.55–0.78)	
≥60	1100	0.82 (0.72–0.93)	
Sex			0.858
Male	633	0.77 (0.65–0.91)	
Female	1014	0.75 (0.66–0.86)	
History of cardiovascular diseases			<0.001
No	404	0.48 (0.38–0.60)	
Yes	1243	0.87 (0.78–0.98)	
History of chronic lower diseases			0.002
No	1397	0.71 (0.63–0.79)	
Yes	250	1.12 (0.86–1.46)	
History of hypertension			0.620
No	374	0.8 (0.64–0.99)	
Yes	482	0.75 (0.67–0.84)	
History of hyperlipidemia			0.270
No	246	0.87 (0.67–1.14)	
Yes	1401	0.74 (0.66–0.83)	
History of ulcer			0.420
No	1509	0.75 (0.67–0.84)	
Yes	138	0.87 (0.61–1.25)	
History of diabetes			0.075
No	1075	0.71 (0.62–0.81)	
Yes	572	0.86 (0.72–1.03)	
History of kidney diseases			0.002
No	1570	0.73 (0.66–0.81)	
Yes	77	1.58 (0.98–2.55)	
History of liver diseases			0.063
No	1134	0.71 (0.63–0.81)	
Yes	513	0.88 (0.73–1.05)	
History of cancer			0.909
No	1599	0.76 (0.68–0.84)	
Yes	48	0.73 (0.4–1.34)	
History of acute respiratory infection^b^			0.986
No	1432	0.72 (0.65–0.80)	
Yes	215	0.72 (0.55–0.95)	
Vaccination frequency^**c**^			0.272
Multiple vaccination	238	0.85 (0.55–1.31)	
Single vaccination	1404	0.70 (0.59–0.84)	

^a^The standard SCCS model was used for stratified analysis, adjusting for acute respiratory infections and seasonal effect. For the stratification factor of whether acute respiratory infection occurred before the acute cardiovascular event during the observation period, only seasonal factors were adjusted. The P value is calculated using the likelihood ratio test, and the null assumption is that the interaction term effect is 0.

^b^ History of acute respiratory infection indicates that acute respiratory infection occurred before the acute cardiovascular event during the observation period.

^c^ If an individual had received another influenza vaccination within one year before the current vaccination, they would be classified as having received multiple vaccinations. Only individuals who received influenza vaccination after September 1, 2016 were included.

In the analysis investigating the impact of vaccination frequency on the prevention of acute cardiovascular events, it was found that the protective effect of multiple vaccination (RI: 0.88, 95% CI: 0.67–1.15) was weaker than that of single vaccination (RI: 0.74, 95% CI: 0.66–0.82). However, no interaction effect between influenza vaccine administration and vaccination frequency was observed (P = 0.272) (Table 2 and Supplementary Table 5).

### Sensitivity analysis

To assess the stability of the results, this study conducted the following sensitivity analysis: not adjusting for acute respiratory infection factors; analyzing different influenza seasons separately; excluding patients who experienced acute respiratory infections and acute cardiovascular events during the same hospitalization period; and excluding patients who died from acute cardiovascular events during hospitalization. The results of sensitivity analysis are consistent with the main analysis results, as shown in Table 3. In addition, to demonstrate the timing and frequency of influenza vaccination among patients with cardiovascular events, we used cardiovascular events as the inclusion criteria and examined their influenza vaccination status (Supplementary Table 6 and Supplementary Table 7).

**Table 3:** Sensitivity analysis for the relative incidence (RI) of acute cardiovascular events following influenza vaccination

		Days 29–84	Days 85–168	Days 169–252	Days 253–365	Days 29–365
Not adjusting for acute respiratory infection	No. of events	112	172	142	203	629
	RI (95% CI)	0.78 (0.64–0.96)	0.78 (0.66–0.93)	0.64 (0.53–0.77)	0.71 (0.6–0.83)	0.72 (0.65–0.80)
Influenza seasons						
Sep 2016–Aug 2017	No. of events	47	51	51	79	228
	RI (95% CI)	1.13 (0.81–1.56)	0.86 (0.62–1.19)	0.96 (0.69–1.34)	0.95 (0.72–1.25)	0.96 (0.81–1.15)
Sep 2017–Aug 2018	No. of events	37	74	66	73	250
	RI (95% CI)	0.97 (0.68–1.38)	1.23 (0.92–1.64)	1.05 (0.77–1.42)	0.89 (0.67–1.19)	1.02 (0.86–1.22)
Other seasons	No. of events	28	47	25	51	151
	RI (95% CI)	0.56 (0.37–0.83)	0.51 (0.37–0.71)	0.25 (0.17–0.39)	0.46 (0.33–0.62)	0.43 (0.36–0.52)
Excluding individuals with acute cardiovascular events and acute respiratory infections in the same hospital stay	No. of events	92	154	128	185	559
	RI (95% CI)	0.74 (0.59–0.93)	0.80 (0.66–0.96)	0.62 (0.51–0.75)	0.70 (0.59–0.82)	0.71 (0.64–0.79)
Excluding deaths during hospitalization for acute cardiovascular events	No. of events	111	171	142	203	627
	RI (95% CI)	0.86 (0.70–1.06)	0.83 (0.70–0.99)	0.67 (0.56–0.82)	0.73 (0.62–0.86)	0.76 (0.69–0.85)

In the analysis exploring the impact of IIV immunity beyond one year, a total of 1360 participants were included (Supplementary Table 8). The results suggest that the protective effect of influenza vaccination on acute cardiovascular events may persist for at least two years. In individuals without a history of cardiovascular diseases, a decline in this protective effect was observed over time (Supplementary Table 9).

## Discussion

The influenza vaccine is a cost-effective strategy for preventing acute respiratory infections and reducing their severity. There exists a complex relationship between acute respiratory infections and acute cardiovascular events, with evidence suggesting that the influenza vaccine also holds great potential for primary and secondary prevention of cardiovascular disease^15,23^. This study aimed to investigate the potential relationship between influenza vaccine and cardiovascular events and provide data support for optimizing strategies for preventing and controlling cardiovascular diseases, a self-controlled case series study based on hospitalization data from Beijing, China, was conducted. The findings revealed that individuals who received the influenza vaccine experienced a 24% reduction in their risk of developing acute cardiovascular events within one year; this protective effect was even more significant among individuals without a history of cardiovascular diseases.

This study has revealed the protective effect of influenza vaccines on acute cardiovascular events. Previous studies have also suggested that influenza vaccines can prevent cardiovascular diseases^6^. A systematic review and meta-analysis showed that influenza vaccines can reduce major adverse cardiovascular events by 13% in individuals with a history of cardiovascular disease^24^. Another SCCS study demonstrated a 27% reduction in hospitalizations for cardiovascular disease among heart failure patients who received influenza vaccines^6^. Our study did not find any differences in the protective effect of the vaccine over different time periods within 1 year, while another similar study indicated a gradual decline in vaccine effectiveness over time^6^. This may be due to the relatively small sample size of our study, leading to wider confidence intervals for estimates of effects at different time points, making it difficult to detect subtle differences between time periods. Furthermore, our findings also indicated that the protective impact of multiple vaccinations was less pronounced compared to single vaccination. This observation is consistent with previous studies which have shown that repeated influenza vaccination attenuate effectiveness^25^.

This study investigated the association between influenza vaccination and acute cardiovascular events, while considering the relationship between acute respiratory infections and acute cardiovascular events. We included acute respiratory infection as an additional exposure factor in addition to influenza vaccination in the model. The findings revealed that the factor of acute respiratory infection essentially does not impact the effect value of influenza vaccine immunization. In order to confirm this conclusion, a stratified analysis based on whether there was an acute respiratory infection before the acute cardiovascular event during the observation period was conducted, along with a likelihood ratio test. The results also showed no statistical difference between the two. According to prior research, it is possible that the association between influenza vaccine and cardiovascular disease may even be stronger than its association with respiratory infections^6,26^. During the observation period of this study, the most frequently detected pathogens were Mycoplasma pneumoniae, Haemophilus influenzae, Klebsiella pneumoniae, influenza A virus, human rhinovirus, and Streptococcus pneumoniae^27,28^. This suggests that the protective mechanism of influenza vaccine against acute cardiovascular events may not solely be achieved through preventing or reducing severe cases of influenza infections but could also be related to non-specific protective effects of vaccines^29^. Several mechanisms have been proposed to explain the protective effect of influenza vaccines on cardiovascular events, independent of their role in preventing influenza infection. For instance, influenza vaccination may activate the immune system, and modulation of immune responses to chronic inflammation could contribute to the cardiovascular benefits observed^30,31^. In addition, influenza vaccination may help stabilize atherosclerotic plaques, thereby reducing the likelihood of plaque rupture and subsequent cardiovascular complications^32^. It is important to note that due to limitations in sample size, particularly for those who had an acute respiratory infection before experiencing an acute cardiovascular event; further validation is still necessary in future studies regarding whether such infections affect the protective effect of influenza vaccine.

This study discovered that the influenza vaccine provides significant protection against myocardial infarction and ischemic stroke among various acute cardiovascular events. However, no association was found between the influenza vaccine and hemorrhagic stroke or other acute cardiovascular events. Several previous studies have confirmed the protective effect of the influenza vaccine on myocardial infarction. For instance, a 14-year cohort study using a case-control design to explore the preventive effect of influenza vaccine on myocardial infarction in elderly individuals found that the vaccine can reduce the risk of myocardial infarction by 39%^33^. A meta-analysis based on a self-controlled case series study showed that within 4 weeks of receiving the influenza vaccine, there is a 16% reduction in the risk of myocardial infarction^7^. However, there is still controversy regarding the association between influenza vaccine and stroke. Some studies have reported that influenza vaccines significantly reduce the risk of ischemic stroke and show a trend of protection against hemorrhagic stroke^34^, while another study indicated that they have little protective effect on strokes in middle-aged individuals^8^. This difference may be due to variances in pathogenesis between myocardial infarction and cerebrovascular disease.

The history of cardiovascular disease is a significant factor that influences the protective effect of vaccines. We found that in individuals without a history of cardiovascular disease, the protective effect is significantly higher compared to those with such a history. However, previous studies have produced inconsistent results regarding the modifying effects of cardiovascular disease history^3,35,36^. This may be influenced by various factors such as study design, follow-up time, and inclusion criteria. The limited protective effect of vaccines on individuals with a history of cardiovascular disease can be attributed to the compromised immunity^37^. Additionally, when patients with a history of cardiovascular disease are hospitalized for certain diseases (such as acute respiratory infections), clinicians may give special attention and timely intervention using antiviral drugs and cardiovascular medications to prevent adverse outcomes^38^. Furthermore, the findings of this study are based on a self-controlled design, in which the relative incidence rate of events during the exposure period and baseline period for individuals was calculated. However, it did not take into account differences between individuals^14,39^. Considering that patients with a history of cardiovascular disease have a higher risk of acute cardiovascular events^40^, individuals with pre-existing heart conditions remain primary focus for intervention through influenza vaccination. Their immunization may have significant implications for public health.

Currently, there is limited evidence regarding the duration of the protective effect of influenza vaccine on cardiovascular events. A study utilizing a self-controlled case series design found that the protective effect of influenza vaccine against acute myocardial infarction can last for 60 days^20^. Another self-controlled case series study investigated the protective effect of influenza vaccine on various etiologies of hospitalization, including cardiovascular diseases, respiratory system diseases, and all-cause hospitalization, and found that the protective effect can last for 300 days, 120 days, and 150 days respectively^6^. Our study demonstrated the protective effect of influenza vaccination on acute cardiovascular events within one-year post-vaccination and we found no decline in vaccine effectiveness over time. The sensitivity analysis of our study suggested that the protective effect of influenza vaccination on acute cardiovascular events may persist for at least two years. The duration of protection may vary in different age groups due to factors such as immunosenescence affecting older individuals or those with multiple comorbidities leading to inadequate immune response following vaccination. Further exploration should be conducted through real-world studies to determine the precise duration of the protective effect of influenza vaccine. If a waning protective effect is confirmed, optimized vaccination strategies such as semi-annual or high-dose vaccinations may be an important solution. These strategies have been shown to have greater advantages in reducing influenza virus infection^41,42^ and related hospitalizations for respiratory system diseases and all causes^43^.

There are several limitations in this study. Firstly, the study population comprised hospitalized individuals in Beijing, predominantly elderly, which may impact the representativeness of the population. Secondly, the exclusion of patients who have been revaccinated within two years may introduce selection bias. The population choosing a single dose may differ systematically from those receiving multiple doses in terms of health status, awareness, and lifestyle. However, we have conducted an analysis to compare the protective effect of multiple vaccination with that of single vaccination and found no statistically significant difference. Therefore, the exclusion of revaccinated individuals may not impact the generalizability of the results. Thirdly, the sample size is relatively small, especially when analyzing different types of cardiovascular events separately, potentially affecting the accuracy of the results. Furthermore, despite employing a self-controlled case series design for analysis, potential confounding factors such as the vaccination of pneumococcal vaccines may still influence the findings.

The study revealed an association between influenza vaccination and a reduced risk of acute cardiovascular events, regardless of individuals’ history of cardiovascular disease. This highlights the potential for influenza vaccination as both a primary and secondary prevention strategy for cardiovascular disease. Additionally, the study found that the protective effect of influenza vaccination against acute cardiovascular events can last for at least 1 year.

## Supplementary information

Supplementary Information

## Data Availability

The data that support the findings of this study are available from Beijing Municipal Health Big Data and Policy Research Center but restrictions apply to the availability of these data, which were used under license for the current study, and so are not publicly available. Data are however available from the authors upon reasonable request and with permission of Beijing Municipal Health Big Data and Policy Research Center.

## Code availability

All code used in this study were available from the corresponding author on reasonable request.
