# Supplementary material for: Effectiveness of influenza vaccines in preventing acute cardiovascular events within 1 year in Beijing, China
Source: NPJ Vaccines. 2024 Sep 28;9:177. doi: 10.1038/s41541-024-00969-y (PMC11438872; doi:10.1038/s41541-024-00969-y)
Supplement: Supplementary file 1 — Supplementary Information [file 41541_2024_969_MOESM1_ESM.docx]

**Supplementary**

**Supplementary Table 1.** Vaccine types during the 2015-2016 to 2018-2019 influenza seasons

**Supplementary Table 2.** Vaccine strains, circulating strains, and their concordance during the 2015-2016 to 2018-2019 influenza seasons in northern China

**Supplementary Table 3.** Diseases defined by discharge diagnosis codes according to the International Classification of Diseases, Tenth Revision (ICD-10)

**Supplementary Table 4.** Relative incidence (RI) of acute cardiovascular events following influenza vaccination

**Supplementary Table 5.** Relative incidence (RI) of acute cardiovascular events following multiple and single vaccinations

**Supplementary Table 6.** Frequency of influenza vaccinations during four influenza seasons (2015-2016, 2016-2017, 2017-2018, 2018-2019) among participants who experienced cardiovascular events from 2016 to 2019

**Supplementary Table 7.** Distribution of influenza vaccinations during the four influenza seasons (2015-2016, 2016-2017, 2017-2018, 2018-2019) in individuals who experienced cardiovascular events from 2016 to 2019

**Supplementary Table 8.** Characteristics of participants in the SCCS study with three-year observation period

**Supplementary Table 9.** Relative incidence (RI) of acute cardiovascular events within two-years following influenza vaccination

**Supplementary Figure 1.** Centered plot for the number of cardiovascular events over time since receiving the influenza vaccine

**Supplementary Table 1. Vaccine types during the 2015-2016 to 2018-2019 influenza seasons**

| **Influenza season** | **Vaccine types** |
| --- | --- |
| 2015-2016 | Trivalent inactivated split-virus influenza vaccine |
| 2016-2017 | Trivalent inactivated split-virus influenza vaccine |
| 2017-2018 | Trivalent inactivated split-virus influenza vaccine;  Trivalent inactivated whole-virus influenza vaccine;  Quadrivalent inactivated split-virus influenza vaccine |
| 2018-2019 | Trivalent inactivated split-virus influenza vaccine;  Trivalent inactivated subunit influenza vaccine;  Quadrivalent inactivated split-virus influenza vaccine |

Note: This information was derived from the data used for analysis in this study.

**Supplementary Table 2. Vaccine strains, circulating strains, and their concordance during the 2015-2016 to 2018-2019 influenza seasons in northern China**

| **Vaccine strains** | **Circulating strains** | **Concordance between vaccine strains and circulating strains** | **Predominant circulating strains** |
| --- | --- | --- | --- |
| **2015-2016 influenza season ^a, b^** |  |  |  |
| an A/California/7/2009 (H1N1)pdm09-like virus | A(H1N1)pdm09 | 98.9% | ✓ |
| an A/Switzerland/9715293/2013 (H3N2)-like virus | A(H3N2) | 80.6% |  |
| a B/Phuket/3073/2013-like virus | B/Yamagata | 98.4% |  |
| - | B/Victoria | - | ✓ |
| **2016-2017 influenza season ^c, d^** |  |  |  |
| an A/California/7/2009 (H1N1)pdm09-like virus | A(H1N1)pdm09 | 92.8% | ✓ |
| an A/Hong Kong/4801/2014 (H3N2)-like virus | A(H3N2) | 41.0% | ✓ |
| a B/Brisbane/60/2008-like virus | B/Victoria | 91.3% |  |
| - | B/Yamagata | - |  |
| **2017-2018 influenza season ^e, f^** |  |  |  |
| an A/Michigan/45/2015 (H1N1)pdm09-like virus | A(H1N1)pdm09 | 94.2% | ✓ |
| an A/Hong Kong/4801/2014 (H3N2)-like virus | A(H3N2) | 34.7% |  |
| a B/Brisbane/60/2008-like virus | B/Victoria | 59.9% |  |
| a B/Phuket/3073/2013-like virus | B/Yamagata | 97.5% | ✓ |
| **2018-2019 influenza seaso**n **^g, h^** |  |  |  |
| an A/Michigan/45/2015 (H1N1)pdm09-like virus | A(H1N1)pdm09 | 97.6% | ✓ |
| an A/Singapore/INFIMH-16-0019/2016 (H3N2)-like virus | A(H3N2) | 78.7% |  |
| a B/Colorado/06/2017-like virus (B/Victoria/2/87 lineage) | B/Victoria | 50.5% |  |
| a B/Phuket/3073/2013-like virus (B/Yamagata/16/88 lineage) | B/Yamagata | 98.3% |  |

^a^ mondiale de la Santé O, World Health Organization. Recommended composition of influenza virus vaccines for use in the 2015-2016 northern hemisphere influenza season[J]. Weekly Epidemiological Record= Relevé épidémiologique hebdomadaire, 2015, 90(11): 97-108.

^b^ CNIC (Chinese National Influenza Center), 2016. Chinese Influenza Weekly Report week 32 2016 [8/23/2016]. Available online: https://ivdc.chinacdc.cn/cnic/en/Surveillance/WeeklyReport/201608/t20160823_133769.htm.

^c^ mondiale de la Santé O, World Health Organization. Recommended composition of influenza virus vaccines for use in the 2016-2017 northern hemisphere influenza season[J]. Weekly Epidemiological Record= Relevé épidémiologique hebdomadaire, 2016, 91(10): 121-132.

^d^ CNIC (Chinese National Influenza Center), 2017. Chinese Influenza Weekly Report week 33 2017 [8/28/2017]. Available online: https://ivdc.chinacdc.cn/cnic/en/Surveillance/WeeklyReport/201708/t20170828_151326.htm.

^e^ mondiale de la Santé O, World Health Organization. Recommended composition of influenza virus vaccines for use in the 2017-2018 northern hemisphere influenza season-Composition recommandée des vaccins antigrippaux pour la saison grippale 2017-2018 dans l’hémisphère Nord[J]. Weekly Epidemiological Record= Relevé épidémiologique hebdomadaire, 2017, 92(11): 117-128.

^f^ CNIC (Chinese National Influenza Center), 2018. Chinese Influenza Weekly Report week 33 2018 [8/24/2018]. Available online: https://ivdc.chinacdc.cn/cnic/en/Surveillance/WeeklyReport/201808/t20180824_189989.htm.

^g^ mondiale de la Santé O, World Health Organization. Recommended composition of influenza virus vaccines for use in the 2018-2019 northern hemisphere influenza season-Composition recommandée des vaccins antigrippaux pour la saison grippale 2018-2019 dans l’hémisphère Nord[J]. Weekly Epidemiological Record= Relevé épidémiologique hebdomadaire, 2018, 93(12): 133-141.

^h^ CNIC (Chinese National Influenza Center), 2019. Chinese Influenza Weekly Report week 34 2019 [8/30/2019]. Available online: https://ivdc.chinacdc.cn/cnic/en/Surveillance/WeeklyReport/201908/t20190830_205138.htm.

**Supplementary Table 3. Diseases defined by discharge diagnosis codes according to the International Classification of Diseases, Tenth Revision (ICD-10)**

| **Types of diseases** | **ICD-10** |
| --- | --- |
| **Acute respiratory infection** |  |
|  | J00–J06 |
|  | J09–J18 |
|  | J20–J22 |
| **Acute cardiovascular diseases** |  |
|  | I20.001 |
|  | I21.001 |
|  | I21.002 |
|  | I21.004 |
|  | I21.005 |
|  | I21.103 |
|  | I21.104 |
|  | I21.105 |
|  | I21.201 |
|  | I21.202 |
|  | I21.203 |
|  | I21.204 |
|  | I21.206 |
|  | I21.207 |
|  | I21.208 |
|  | I21.209 |
|  | I21.210 |
|  | I21.211 |
|  | I21.214 |
|  | I21.215 |
|  | I21.216 |
|  | I21.217 |
|  | I21.218 |
|  | I21.219 |
|  | I21.220 |
|  | I21.221 |
|  | I21.222 |
|  | I21.223 |
|  | I21.224 |
|  | I21.225 |
|  | I21.226 |
|  | I21.227 |
|  | I21.228 |
|  | I21.229 |
|  | I21.230 |
|  | I21.304 |
|  | I21.401 |
|  | I21.402 |
|  | I21.403 |
|  | I21.404 |
|  | I21.901 |
|  | I21.902 |
|  | I22.001 |
|  | I22.002 |
|  | I22.003 |
|  | I22.101 |
|  | I22.102 |
|  | I22.103 |
|  | I22.801 |
|  | I22.802 |
|  | I22.803 |
|  | I22.804 |
|  | I22.805 |
|  | I22.806 |
|  | I22.807 |
|  | I22.808 |
|  | I22.809 |
|  | I22.810 |
|  | I22.811 |
|  | I22.812 |
|  | I22.813 |
|  | I22.814 |
|  | I22.815 |
|  | I22.816 |
|  | I22.817 |
|  | I22.818 |
|  | I22.901 |
|  | I24.001 |
|  | I24.002 |
|  | I24.003 |
|  | I24.802 |
|  | I24.803 |
|  | I24.804 |
|  | I24.901 |
|  | I30.001 |
|  | I30.901 |
|  | I30.902 |
|  | I30.903 |
|  | I40.005 |
|  | I40.901 |
|  | I50.006 |
|  | I50.102 |
|  | I50.904 |
|  | I60.001 |
|  | I60.101 |
|  | I60.201 |
|  | I60.301 |
|  | I60.401 |
|  | I60.501 |
|  | I60.807 |
|  | I60.901 |
|  | I61.001 |
|  | I61.002 |
|  | I61.003 |
|  | I61.004 |
|  | I61.005 |
|  | I61.006 |
|  | I61.007 |
|  | I61.008 |
|  | I61.009 |
|  | I61.010 |
|  | I61.011 |
|  | I61.101 |
|  | I61.102 |
|  | I61.103 |
|  | I61.104 |
|  | I61.105 |
|  | I61.106 |
|  | I61.107 |
|  | I61.108 |
|  | I61.109 |
|  | I61.110 |
|  | I61.111 |
|  | I61.112 |
|  | I61.113 |
|  | I61.114 |
|  | I61.115 |
|  | I61.201 |
|  | I61.301 |
|  | I61.302 |
|  | I61.303 |
|  | I61.304 |
|  | I61.401 |
|  | I61.402 |
|  | I61.403 |
|  | I61.501 |
|  | I61.502 |
|  | I61.503 |
|  | I61.504 |
|  | I61.505 |
|  | I61.601 |
|  | I61.902 |
|  | I61.903 |
|  | I63.001 |
|  | I63.101 |
|  | I63.201 |
|  | I63.301 |
|  | I63.302 |
|  | I63.401 |
|  | I63.402 |
|  | I63.501 |
|  | I63.502 |
|  | I63.601 |
|  | I63.901 |
|  | I63.902 |
|  | I63.904 |
|  | I63.905 |
|  | I63.906 |
|  | I63.907 |
|  | I63.908 |
|  | I63.909 |
|  | I64xx01 |
|  | I64xx02 |
| **Disease history in this study** |  |
| Cardiovascular diseases | I20–I25, I60–I69, Z86.7, excluding I63.903, I63.81 |
| Hypertension | I10–I15 |
| Hyperlipidemia | E78 |
| Ulcer | K25–K28 |
| Diabetes | E10–E14 |
| Chronic lower respiratory diseases | J40–J47 |
| Kidney diseases | N18, I12, I13, E10.2, E11.2, Z49.0, Z49.1, Z99.1 |
| Liver diseases | K70–K77 |
| Cancer | C00–C97 |

**Supplementary Table 4. Relative incidence (RI) of acute cardiovascular events following influenza vaccination**

|  | **Total** | |  | **With a history of cardiovascular diseases** | |  | **Without a history of cardiovascular diseases** | |
| --- | --- | --- | --- | --- | --- | --- | --- | --- |
|  | **No. of events^*^** | **RI (95% CI)** |  | **No. of events** | **RI (95% CI)** |  | **No. of events** | **RI (95% CI)** |
| **Total events** |  |  |  |  |  |  |  |  |
| Days 29–84 | 112 | 0.86  (0.7–1.06) |  | 102 | 1.09  (0.87–1.35) |  | 10 | 0.28  (0.15–0.53) |
| Days 85–168 | 172 | 0.83  (0.69–0.98) |  | 142 | 0.96  (0.79–1.17) |  | 30 | 0.49  (0.33–0.73) |
| Days 169–252 | 142 | 0.67  (0.55–0.81) |  | 113 | 0.76  (0.62–0.95) |  | 29 | 0.45  (0.30–0.67) |
| Days 253–365 | 203 | 0.73  (0.62–0.86) |  | 159 | 0.79  (0.66–0.96) |  | 44 | 0.57  (0.40–0.80) |
| Days 29–365 | 629 | 0.76  (0.68–0.84) |  | 516 | 0.88  (0.78–0.99) |  | 113 | 0.47  (0.38–0.59) |
| Days 0–28, 366–730  (Control period) | 1018 | 1.00 |  | 727 | 1.00 |  | 291 | 1.00 |
| **Ischemic stroke** |  |  |  |  |  |  |  |  |
| Days 29–84 | 28 | 0.55  (0.37–0.83) |  | 24 | 1.09  (0.69–1.72) |  | 4 | 0.14  (0.05–0.39) |
| Days 85–168 | 50 | 0.61  (0.45–0.84) |  | 27 | 0.84  (0.54–1.32) |  | 23 | 0.47  (0.3–0.73) |
| Days 169–252 | 50 | 0.55  (0.40–0.75) |  | 29 | 0.82  (0.53–1.27) |  | 21 | 0.38  (0.24–0.61) |
| Days 253–365 | 69 | 0.59  (0.45–0.78) |  | 32 | 0.63  (0.42–0.95) |  | 37 | 0.58  (0.4–0.85) |
| Days 29–365 | 197 | 0.58  (0.49–0.69) |  | 112 | 0.8  (0.62–1.03) |  | 85 | 0.43  (0.34–0.56) |
| Days 0–28, 366–730  (Control period) | 403 | 1.00 |  | 166 | 1.00 |  | 237 | 1.00 |
| **Hemorrhagic stroke** |  |  |  |  |  |  |  |  |
| Days 29–84 | 8 | 1.33  (0.59–2.96) |  | 2 | 1.15  (0.24–5.51) |  | 6 | 1.36  (0.53–3.46) |
| Days 85–168 | 7 | 0.74  (0.31–1.77) |  | 4 | 1.57  (0.41–6.04) |  | 3 | 0.43  (0.12–1.5) |
| Days 169–252 | 5 | 0.69  (0.25–1.92) |  | 0 | 0  (0–Inf) |  | 5 | 1.07  (0.36–3.15) |
| Days 253–365 | 6 | 0.53  (0.21–1.34) |  | 1 | 0.32  (0.04–2.73) |  | 5 | 0.63  (0.23–1.77) |
| Days 29–365 | 26 | 0.78  (0.46–1.3) |  | 7 | 0.72  (0.26–1.97) |  | 19 | 0.8  (0.44–1.45) |
| Days 0–28, 366–730  (Control period) | 42 | 1.00 |  | 12 | 1.00 |  | 30 | 1.00 |
| **Myocardial infarction** |  |  |  |  |  |  |  |  |
| Days 29–84 | 11 | 0.65  (0.34–1.23) |  | 11 | 0.66  (0.35–1.26) |  | 0 | – |
| Days 85–168 | 20 | 0.71  (0.43–1.17) |  | 20 | 0.73  (0.44–1.22) |  | 0 | – |
| Days 169–252 | 20 | 0.74  (0.45–1.24) |  | 18 | 0.71  (0.42–1.22) |  | 2 | – |
| Days 253–365 | 26 | 0.80  (0.50–1.27) |  | 25 | 0.80  (0.5–1.29) |  | 1 | – |
| Days 29–365 | 77 | 0.74  (0.55–0.99) |  | 74 | 0.74  (0.55–1) |  | 3 | – |
| Days 0–28, 366–730  (Control period) | 132 | 1.00 |  | 127 | 1.00 |  | 5 | – |
| **Other acute cardiovascular events** |  |  |  |  |  |  |  |  |
| Days 29–84 | 65 | 1.18  (0.89–1.55) |  | 65 | 1.22  (0.93–1.62) |  | 0 | 0  (0–Inf) |
| Days 85–168 | 93 | 1.07  (0.84–1.37) |  | 89 | 1.05  (0.82–1.36) |  | 4 | 1.62  (0.39–6.69) |
| Days 169–252 | 66 | 0.77  (0.58–1.02) |  | 66 | 0.79  (0.6–1.05) |  | 0 | 0  (0–Inf) |
| Days 253–365 | 101 | 0.86  (0.68–1.09) |  | 100 | 0.88  (0.69–1.11) |  | 1 | 0.32  (0.04–2.65) |
| Days 29–365 | 325 | 0.95  (0.81–1.1) |  | 320 | 0.96  (0.83–1.12) |  | 5 | 0.47  (0.15–1.43) |
| Days 0–28, 366–730  (Control period) | 429 | 1.00 |  | 415 | 1.00 |  | 14 | 1.00 |

**^*^** For the analysis of acute cardiovascular disease subtypes, individuals with a discharge diagnosis containing only that specific type of acute cardiovascular disease were included. Participants with multiple types of events occurring simultaneously were excluded from the study.

**Supplementary Table 5. Relative incidence (RI) of acute cardiovascular events following multiple and single vaccinations**

|  | **Total** | |  | **Multiple vaccination** | |  | **Single vaccination** | | **P value^*^** |
| --- | --- | --- | --- | --- | --- | --- | --- | --- | --- |
|  | **No. of events** | **RI (95% CI)** |  | **No. of events** | **RI (95% CI)** |  | **No. of events** | **RI (95% CI)** |  |
| Days 29–365 | 627 | 0.76 (0.68-0.84) |  | 99 | 0.88 (0.67-1.15) |  | 528 | 0.74 (0.66-0.82) | 0.272 |
| Days 29–84 | 110 | 0.84 (0.69-1.04) |  | 15 | 0.72 (0.42-1.25) |  | 95 | 0.87 (0.69-1.08) | - |
| Days 85–168 | 172 | 0.83 (0.69-0.99) |  | 25 | 0.8 (0.50-1.28) |  | 147 | 0.83 (0.68-1) | - |
| Days 169–252 | 142 | 0.67 (0.55-0.81) |  | 28 | 1.19 (0.74-1.93) |  | 114 | 0.61 (0.49-0.75) | - |
| Days 253–365 | 203 | 0.72 (0.61-0.85) |  | 31 | 0.85 (0.55-1.31) |  | 172 | 0.7 (0.59-0.84) | - |
| Days 0–28, 366–730  (Control period) | 1015 | 1.00 |  | 139 | 1.00 |  | 876 | 1.00 | - |

* The P-value is calculated using the likelihood ratio test, and the null assumption is that the interaction term effect is 0.

**Supplementary Table 6. Frequency of influenza vaccinations during four influenza seasons (2015-2016, 2016-2017, 2017-2018, 2018-2019) among participants who experienced cardiovascular events from 2016 to 2019**

|  | **n** | **Percent (%)** |
| --- | --- | --- |
| N | 315580 | 100.0 |
| Frequency, Mean±SD | 1.95±1.09 | - |
| None | 302633 | 4.10 |
| One time | 6264 | 48.38 |
| Two times | 2842 | 21.95 |
| Three times | 2027 | 15.66 |
| Four times | 1814 | 14.01 |

**Supplementary Table 7. Distribution of influenza vaccinations during the four influenza seasons (2015-2016, 2016-2017, 2017-2018, 2018-2019) in individuals who experienced cardiovascular events from 2016 to 2019**

| Frequency of influenza vaccinations | 2015-2016 | 2016-2017 | 2017-2018 | 2018-2019 | n (%) |
| --- | --- | --- | --- | --- | --- |
| None |  |  |  |  | 302633 (95.90) |
| One time | ✓ |  |  |  | 2389 (0.76) |
|  |  | ✓ |  |  | 967 (0.31) |
|  |  |  | ✓ |  | 1208 (0.38) |
|  |  |  |  | ✓ | 1700 (0.54) |
| Two times | ✓ | ✓ |  |  | 1051 (0.33) |
|  | ✓ |  | ✓ |  | 379 (0.12) |
|  | ✓ |  |  | ✓ | 221 (0.07) |
|  |  | ✓ | ✓ |  | 405 (0.13) |
|  |  | ✓ |  | ✓ | 172 (0.05) |
|  |  |  | ✓ | ✓ | 614 (0.19) |
| Three times | ✓ | ✓ | ✓ |  | 955 (0.30) |
|  | ✓ | ✓ |  | ✓ | 293 (0.09) |
|  | ✓ |  | ✓ | ✓ | 320 (0.10) |
|  |  | ✓ | ✓ | ✓ | 459 (0.15) |
| Four times | ✓ | ✓ | ✓ | ✓ | 1814 (0.57) |

**Supplementary Table 8. Characteristics of participants in the SCCS study with three-year observation period**

|  | **Total events***  **(N=1360)** | **Myocardial infarction**  **(N=173)** | **Ischemic stroke**  **(N=506)** | **Hemorrhagic stroke**  **(N=59)** | **Others**  **(N=610)** |
| --- | --- | --- | --- | --- | --- |
| **Age (Years), median (Q1-Q3)** | 65.00 (56.00-74.00) | 63.00 (52.00-73.00) | 67.00 (58.00-75.00) | 58.00 (50.00-73.00) | 65.50 (56.00-73.00) |
| **Age group (Years), n (%)** |  |  |  |  |  |
| <60 | 447 (32.87) | 68 (39.31) | 145 (28.66) | 30 (50.85) | 200 (32.79) |
| ≥60 | 913 (67.13) | 105 (60.69) | 361 (71.34) | 29 (49.15) | 410 (67.21) |
| **Sex, n (%)** |  |  |  |  |  |
| Male | 834 (61.32) | 124 (71.68) | 326 (64.43) | 37 (62.71) | 338 (55.41) |
| Female | 526 (38.68) | 49 (28.32) | 180 (35.57) | 22 (37.29) | 272 (44.59) |
| **Length of hospital stay (Days), median (Q1-Q3)** | 8.00 (6.00-12.00) | 8.00 (6.00-11.00) | 10.00 (8.00-13.00) | 13.00 (9.00-18.00) | 7.00 (4.00-10.00) |
| **Acute respiratory infection occurred^**^, n (%)** | 192 (14.12) | 29 (16.76) | 74 (14.62) | 4 (6.78) | 82 (13.44) |
| **Underlying diseases, n (%)** |  |  |  |  |  |
| Cardiovascular diseases | 1022 (75.15) | 167 (96.53) | 233 (46.05) | 15 (25.42) | 597 (97.87) |
| Chronic lower respiratory infection | 197 (14.49) | 13 (7.51) | 76 (15.02) | 5 (8.47) | 103 (16.89) |
| Hypertension | 1045 (76.84) | 110 (63.58) | 407 (80.43) | 49 (83.05) | 468 (76.72) |
| Hyperlipidemia | 1151 (84.63) | 149 (86.13) | 451 (89.13) | 24 (40.68) | 515 (84.43) |
| Ulcer | 113 (8.31) | 19 (10.98) | 41 (8.10) | 11 (18.64) | 40 (6.56) |
| Diabetes | 489 (35.96) | 56 (32.37) | 205 (40.51) | 8 (13.56) | 216 (35.41) |
| Kidney disease | 70 (5.15) | 7 (4.05) | 33 (6.52) | 1 (1.69) | 29 (4.75) |
| Liver diseases | 435 (31.99) | 48 (27.75) | 154 (30.43) | 12 (20.34) | 216 (35.41) |
| Cancer | 39 (2.87) | 7 (4.05) | 16 (3.16) | 2 (3.39) | 14 (2.30) |

* There were an additional 12 participants who experienced at least two types of diseases within the three categories of myocardial infarction, ischemic stroke, and hemorrhagic stroke.

** Occurring acute respiratory infection before the first acute cardiovascular event during the observation period.

**Supplementary Table 9. Relative incidence (RI) of acute cardiovascular events within two-years following influenza vaccination ^*^**

|  | **Total^**^** | |  | **With a history of cardiovascular diseases** | |  | **Without a history of cardiovascular diseases** | |
| --- | --- | --- | --- | --- | --- | --- | --- | --- |
|  | **No. of events** | **RI (95% CI)** |  | **No. of events** | **RI (95% CI)** |  | **No. of events** | **RI (95% CI)** |
| **Total events** |  |  |  |  |  |  |  |  |
| Days 29–90 | 59 | 0.60 (0.45-0.79) |  | 55 | 0.78 (0.58-1.04) |  | 4 | 0.14 (0.05-0.39) |
| Days 91–181 | 90 | 0.55 (0.44-0.69) |  | 71 | 0.62 (0.48-0.81) |  | 19 | 0.38 (0.23-0.63) |
| Days 182–365 | 160 | 0.47 (0.39-0.56) |  | 129 | 0.52 (0.42-0.64) |  | 31 | 0.34 (0.23-0.51) |
| Days 366–456 | 98 | 0.6 (0.48-0.76) |  | 71 | 0.59 (0.45-0.77) |  | 27 | 0.65 (0.42-1.00) |
| Days 457–547 | 91 | 0.53 (0.42-0.67) |  | 69 | 0.57 (0.44-0.75) |  | 22 | 0.43 (0.27-0.68) |
| Days 548–638 | 90 | 0.52 (0.42-0.66) |  | 69 | 0.57 (0.43-0.74) |  | 21 | 0.42 (0.26-0.67) |
| Days 639–730 | 92 | 0.57 (0.46-0.72) |  | 67 | 0.57 (0.43-0.74) |  | 25 | 0.61 (0.39-0.95) |
| Days 29–730 | 680 | 0.54 (0.48-0.60) |  | 531 | 0.58 (0.51-0.66) |  | 149 | 0.42 (0.34-0.53) |
| Days 0–28, 731–1095  (Control period) | 680 | 1.00 |  | 491 | 1.00 |  | 189 | 1.00 |
| **Ischemic stroke** |  |  |  |  |  |  |  |  |
| Days 29–90 | 14 | 0.34 (0.2-0.6) |  | 13 | 0.74 (0.4-1.35) |  | 1 | 0.05 (0.01-0.32) |
| Days 91–181 | 30 | 0.45 (0.3-0.66) |  | 16 | 0.6 (0.34-1.06) |  | 14 | 0.36 (0.2-0.63) |
| Days 182–365 | 50 | 0.4 (0.29-0.56) |  | 28 | 0.61 (0.39-0.97) |  | 22 | 0.29 (0.18-0.47) |
| Days 366–456 | 26 | 0.43 (0.28-0.66) |  | 10 | 0.38 (0.19-0.74) |  | 16 | 0.49 (0.29-0.85) |
| Days 457–547 | 43 | 0.65 (0.46-0.92) |  | 23 | 0.93 (0.57-1.52) |  | 20 | 0.48 (0.29-0.79) |
| Days 548–638 | 41 | 0.67 (0.47-0.95) |  | 21 | 1.02 (0.61-1.7) |  | 20 | 0.48 (0.29-0.78) |
| Days 639–730 | 44 | 0.78 (0.56-1.1) |  | 20 | 0.96 (0.57-1.6) |  | 24 | 0.68 (0.43-1.07) |
| Days 29–730 | 248 | 0.53 (0.44-0.63) |  | 131 | 0.72 (0.55-0.95) |  | 117 | 0.41 (0.32-0.52) |
| Days 0–28, 731–1095  (Control period) | 258 | 1.00 |  | 102 | 1.00 |  | 156 | 1.00 |
| **Myocardial infarction** |  |  |  |  |  |  |  |  |
| Days 29–90 | 5 | 0.39 (0.16-0.98) |  | 5 | 0.39 (0.16-0.99) |  | 0 | - |
| Days 91–181 | 9 | 0.4 (0.2-0.83) |  | 9 | 0.41 (0.2-0.85) |  | 0 | - |
| Days 182–365 | 25 | 0.46 (0.29-0.74) |  | 22 | 0.41 (0.25-0.68) |  | 3 | - |
| Days 366–456 | 12 | 0.54 (0.29-1.03) |  | 12 | 0.54 (0.28-1.03) |  | 0 | - |
| Days 457–547 | 8 | 0.33 (0.16-0.7) |  | 7 | 0.3 (0.13-0.66) |  | 1 | - |
| Days 548–638 | 10 | 0.37 (0.18-0.72) |  | 10 | 0.38 (0.19-0.75) |  | 0 | - |
| Days 639–730 | 3 | 0.12 (0.04-0.38) |  | 3 | 0.12 (0.04-0.38) |  | 0 | - |
| Days 29–730 | 72 | 0.38 (0.27-0.52) |  | 68 | 0.36 (0.26-0.5) |  | 4 | - |
| Days 0–28, 731–1095  (Control period) | 101 | 1.00 |  | 99 | 1.00 |  | 2 | - |
| **Other acute cardiovascular events** |  |  |  |  |  |  |  |  |
| Days 29–90 | 36 | 0.92 (0.64-1.33) |  | 36 | 0.94 (0.65-1.35) |  | 0 | - |
| Days 91–181 | 46 | 0.73 (0.52-1.01) |  | 44 | 0.7 (0.5-0.98) |  | 2 | - |
| Days 182–365 | 77 | 0.52 (0.4-0.68) |  | 77 | 0.53 (0.41-0.7) |  | 0 | - |
| Days 366–456 | 51 | 0.74 (0.54-1.02) |  | 46 | 0.68 (0.49-0.95) |  | 5 | - |
| Days 457–547 | 40 | 0.59 (0.41-0.83) |  | 39 | 0.57 (0.4-0.81) |  | 1 | - |
| Days 548–638 | 38 | 0.52 (0.36-0.74) |  | 37 | 0.51 (0.36-0.73) |  | 1 | - |
| Days 639–730 | 41 | 0.58 (0.41-0.81) |  | 41 | 0.59 (0.42-0.83) |  | 0 | - |
| Days 29–730 | 329 | 0.62 (0.53-0.74) |  | 320 | 0.62 (0.52-0.73) |  | 9 | - |
| Days 0–28, 731–1095  (Control period) | 281 | 1.00 |  | 277 | 1.00 |  | 4 | - |

**^*^** For the analysis of acute cardiovascular disease subtypes, individuals with a discharge diagnosis containing only that specific type of acute cardiovascular disease were included. Participants with multiple types of events occurring simultaneously were excluded from the study. The results for hemorrhagic stroke were not shown because of the limited sample size.

^**^ Only individuals who received the influenza vaccination between January 1, 2016, and December 31, 2017, and did not receive any subsequent vaccinations within three years were included in the study.


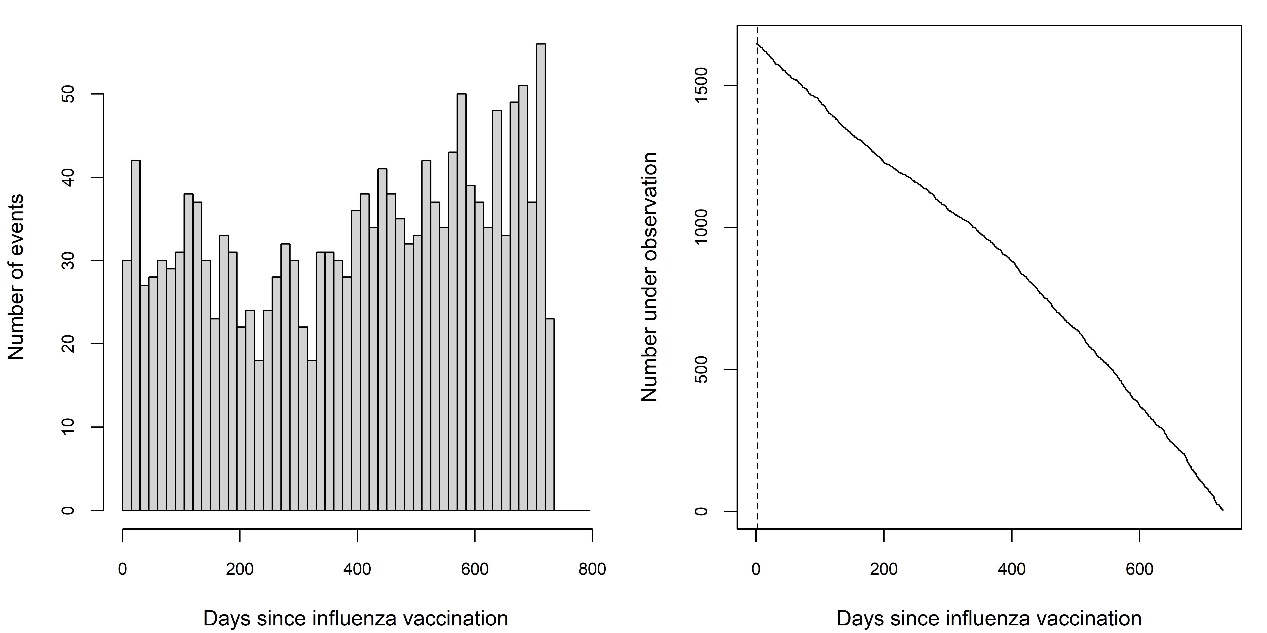


**Supplementary Figure 1. Centered plot for the number of cardiovascular events over time since receiving the influenza vaccine**
